# PGS-GS: a framework integrating polygenic scores and genomic selection in animal breeding

**DOI:** 10.1093/bib/bbag397

**Published:** 2026-09-01

**Authors:** Jinbu Wang, Lili Du, Zhida Zhao, Li Qian, Keanning Li, Shiyuan Qiu, Meng Mao, Mang Liang, Zezhao Wang, Hongwei Li, Yan Chen, Bo Zhu, Caihong Zheng, Xue Gao, Lingyang Xu, Lupei Zhang, Junya Li, Huijiang Gao

**Affiliations:** State Key Laboratory of Animal Biotech Breeding, Institute of Animal Science, Chinese Academy of Agricultural Sciences, No. 2 Yuanmingyuan West Road, Haidian District, Beijing 100193, China; State Key Laboratory of Animal Biotech Breeding, Institute of Animal Science, Chinese Academy of Agricultural Sciences, No. 2 Yuanmingyuan West Road, Haidian District, Beijing 100193, China; State Key Laboratory of Animal Biotech Breeding, Institute of Animal Science, Chinese Academy of Agricultural Sciences, No. 2 Yuanmingyuan West Road, Haidian District, Beijing 100193, China; State Key Laboratory of Animal Biotech Breeding, Institute of Animal Science, Chinese Academy of Agricultural Sciences, No. 2 Yuanmingyuan West Road, Haidian District, Beijing 100193, China; State Key Laboratory of Animal Biotech Breeding, Institute of Animal Science, Chinese Academy of Agricultural Sciences, No. 2 Yuanmingyuan West Road, Haidian District, Beijing 100193, China; State Key Laboratory of Animal Biotech Breeding, Institute of Animal Science, Chinese Academy of Agricultural Sciences, No. 2 Yuanmingyuan West Road, Haidian District, Beijing 100193, China; State Key Laboratory of Animal Biotech Breeding, Institute of Animal Science, Chinese Academy of Agricultural Sciences, No. 2 Yuanmingyuan West Road, Haidian District, Beijing 100193, China; State Key Laboratory of Animal Biotech Breeding, Institute of Animal Science, Chinese Academy of Agricultural Sciences, No. 2 Yuanmingyuan West Road, Haidian District, Beijing 100193, China; State Key Laboratory of Animal Biotech Breeding, Institute of Animal Science, Chinese Academy of Agricultural Sciences, No. 2 Yuanmingyuan West Road, Haidian District, Beijing 100193, China; State Key Laboratory of Animal Biotech Breeding, Institute of Animal Science, Chinese Academy of Agricultural Sciences, No. 2 Yuanmingyuan West Road, Haidian District, Beijing 100193, China; State Key Laboratory of Animal Biotech Breeding, Institute of Animal Science, Chinese Academy of Agricultural Sciences, No. 2 Yuanmingyuan West Road, Haidian District, Beijing 100193, China; State Key Laboratory of Animal Biotech Breeding, Institute of Animal Science, Chinese Academy of Agricultural Sciences, No. 2 Yuanmingyuan West Road, Haidian District, Beijing 100193, China; State Key Laboratory of Animal Biotech Breeding, Institute of Animal Science, Chinese Academy of Agricultural Sciences, No. 2 Yuanmingyuan West Road, Haidian District, Beijing 100193, China; State Key Laboratory of Animal Biotech Breeding, Institute of Animal Science, Chinese Academy of Agricultural Sciences, No. 2 Yuanmingyuan West Road, Haidian District, Beijing 100193, China; State Key Laboratory of Animal Biotech Breeding, Institute of Animal Science, Chinese Academy of Agricultural Sciences, No. 2 Yuanmingyuan West Road, Haidian District, Beijing 100193, China; State Key Laboratory of Animal Biotech Breeding, Institute of Animal Science, Chinese Academy of Agricultural Sciences, No. 2 Yuanmingyuan West Road, Haidian District, Beijing 100193, China; State Key Laboratory of Animal Biotech Breeding, Institute of Animal Science, Chinese Academy of Agricultural Sciences, No. 2 Yuanmingyuan West Road, Haidian District, Beijing 100193, China; State Key Laboratory of Animal Biotech Breeding, Institute of Animal Science, Chinese Academy of Agricultural Sciences, No. 2 Yuanmingyuan West Road, Haidian District, Beijing 100193, China

**Keywords:** genomic selection, polygenic scores, linkage disequilibrium, machine learning, weighting

## Abstract

Genomic prediction has become a central paradigm in biology, enabling quantitative inference of genetic contributions to complex traits across humans, animals, and plants. Although genomic research in human genetics and animal breeding shares a highly homologous methodological foundation, significant barriers persist in their analytical paradigms and application scenarios. This study aims to promote cross-disciplinary integration by introducing human-derived polygenic scores (PGS) algorithms into animal genomic selection (GS) and proposing a PGS-GS framework with a preliminary weighting-based implementation. We systematically benchmarked the predictive performance and computational efficiency of 20 algorithms, including classical linear models, machine learning, PGS, and PGS-GS using both array and whole-genome sequencing (WGS) data across four major agricultural species: beef cattle, sheep, pigs, and chickens. Our results demonstrate that PGS and PGS–GS algorithms achieve predictive accuracy competitive with genomic best linear unbiased prediction (GBLUP) while offering markedly higher computational efficiency. Moreover, incorporating PGS-derived prior information into weighted linear and non-linear models outperformed conventional weighted GBLUP. The results provide empirical evidence to inform algorithm selection and highlight the potential of integrating human-derived PGS methodologies into animal genomic prediction frameworks.

## Introduction

Genomic prediction has become central to human, animal, and plant biology, enabling quantitative inference of how genetic variation shapes complex traits [1]. In human genetics, polygenic scores (PGS), often referred to as polygenic risk scores (PRS), have emerged as promising tools for predicting disease risk and clinical outcomes [2]. Yet many of the core concepts behind these methods, including the use of genome-wide single-nucleotide polymorphisms (SNPs) to estimate genetic effects, originated from animal breeding. This shared foundation illustrates how advances in one field can help to shape another.

Complex traits are typically governed by a highly polygenic architecture with predominantly small-effect variants [3]. Historically, the stringent single-locus significance thresholds employed in traditional genome-wide association studies (GWAS) resulted in the omission of numerous causal loci, culminating in the well-documented “missing heritability” phenomenon [4]. In 2001, Meuwissen *et al.* proposed genomic selection (GS), with the core assumption that all quantitative trait loci (QTL) are in linkage disequilibrium (LD) with at least one marker across the whole genome [5]. This holistic framework, which relies on the joint estimation of marker effects across the entire genome, revolutionized agricultural breeding programs and catalyzed substantial genetic gains, initially in dairy cattle [6, 7]. Concurrently, human genetics underwent a parallel conceptual evolution. In 2009, the International Schizophrenia Consortium (ISC) demonstrated that aggregating sub-threshold variants in LD with causal mutations could robustly predict complex disease risk, thereby laying the foundation for PGS [8]. Subsequently, Yang *et al.* adapted the genomic relationship matrix (GRM) from the GS framework, providing statistical confirmation that missing heritability is predominantly driven by common, small-effect genome-wide variants [9, 10]. Since then, PGS has emerged as a cornerstone of human precision medicine, driving large-scale population genomic initiatives and the mechanistic parsing of complex diseases.

From a methodological perspective, best linear unbiased prediction (BLUP) and Bayesian frameworks constitute the foundational statistical pillars of both GS and PGS [1, 11]. In animal breeding, the genomic BLUP (GBLUP) model, which posits a uniform contribution of all SNPs to genetic variance, has been widely adopted as the industry standard due to its computational efficiency and robustness [12]. Conversely, a diverse array of Bayesian models has been developed to capture the heterogeneous and often sparse genetic architectures of various traits by specifying distinct prior distributions [5, 13, 14]. In the human genetics domain, the necessity to process summary statistics from massive cohorts and navigate intricate, whole-genome LD structures has driven the evolution of highly sophisticated predictive algorithms. Transitioning from traditional clumping and thresholding (C + T) method [3, 15], modern PGS models have increasingly adopted penalized regression and Bayesian architectures [16]. These advanced algorithms can deeply dissect local LD matrices, enabling the highly precise joint estimation of variant effects [17]. Nevertheless, both classical GS and PGS models are fundamentally constrained by their linear assumptions [18, 19]. These linear frameworks restrict their capacity to capture non-additive genetic variance, such as epistasis and dominance, and complex genotype-by-environment interactions [20]. To resolve these issues, machine learning (ML) is being rapidly introduced. Leveraging a flexible architecture free from stringent statistical assumptions, ML can autonomously resolve non-linear interactions, thereby opening a completely new pathway to enhance the prediction accuracy for complex traits [21].

Integrating the differential genetic contributions of diverse loci via marker-specific weighting has emerged as a pivotal strategy to enhance genomic prediction accuracy [22–24]. For instance, Zhang *et al.* proposed the trait-specific marker-derived relationship matrix, which utilizes marker-specific genetic variances as weights to more accurately construct the variance–covariance structure tailored to a given trait [25]. In 2024, Meuwissen *et al.* introduced the genome-wide association assisted best linear unbiased prediction (GWABLUP), which combines smoothed GWAS likelihood ratios with prior probabilities to estimate posterior marker weights, yielding improved prediction reliabilities for dairy cattle traits [22]. As predictive modeling continues to evolve, the application of weighting strategies has transcended linear frameworks, extending into the domain of non-parametric ML. Notably, Diao *et al.* demonstrated that incorporating GWAS-derived *P*-values as locus-specific weights into non-linear frameworks like kernel ridge regression (KRR) significantly enhances the ability to capture non-additive genetic architectures [26]. Collectively, these methodological advances underscore the critical utility of weighting strategies in optimizing predictive performance.

This study aims to promote the integration of human genetics and animal breeding. By introducing PGS methodologies into animal GS, we evaluated the performance of cross-disciplinary prediction models across diverse agricultural species. Within this context, we proposed a PGS–GS framework and implemented a preliminary weighting-based strategy for single-trait genomic prediction. To comprehensively evaluate the effectiveness and generalizability of this interdisciplinary strategy, we systematically benchmarked 20 prediction models using datasets from four major agricultural species: beef cattle, pigs, chickens, and sheep. In the pig and beef cattle datasets, we further compared the performance of different PGS algorithms and prediction models using SNP arrays and whole-genome sequencing (WGS) data. Overall, this study provides empirical insights into the transferability of human genetic methodologies to animal breeding and establishes a foundation for future extensions to multi-trait, multi-breed, and cross-population genomic prediction.

## Materials and methods

### PGS methods

#### PRSice2 (C + T)

PRSice2 implements the C + T method [27]. It first performs LD-driven clumping by iteratively removing SNPs in high LD with the most significant index SNP within a specified genomic window. Subsequently, multiple *P*-value thresholds are applied to the remaining SNPs to identify the parameter combination yielding the best prediction performance.

#### LDpred2

LDpred2 [28] is an advanced computational extension of the LDpred [29] method. It estimates the posterior mean of SNP effects based on GWAS summary statistics and an external LD reference panel, assuming a Gaussian spike-and-slab prior for the effect size ${\beta}_j$:


$${\beta}_j\sim \left\{\begin{array}{@{}ll@{}}N\left(0,\frac{h^2}{mp}\right) & \mathrm{with}\ \mathrm{probability}\ p\\{}0 & \mathrm{with}\ \mathrm{probability}\ 1-p\end{array}\right.,$$

where ${h}^2$ is the total heritability, *m* is the total number of SNPs, and *p* represents the proportion of causal variants. Gibbs sampling is then employed to estimate posterior SNP effects conditional on the marginal GWAS effects and the sparse banded LD matrix.

#### Lassosum2

Lassosum2 [30] is an extension of the lassosum [31] method. To ensure the convexity of the optimization problem when GWAS and LD data are derived from different cohorts, it introduces a shrinkage parameter *s* to construct a regularized LD matrix ${\mathbf{R}}_s=\left(1-s\right)\mathbf{R}+s\mathbf{I}$. The objective function is defined as:


$$f\left(\beta \right)={\beta}^{\mathrm{T}}{\mathbf{R}}_s\beta -2{\beta}^{\mathrm{T}}\mathbf{r}+2\lambda \parallel \beta{\parallel}_1$$

where $\mathbf{R}$ is the LD correlation matrix, $\mathbf{r}$ is the vector of marginal SNP-phenotype correlations, *λ* is the L1 penalty parameter, and $s\in \left(0,1\right)$. Lassosum2 further improves computational efficiency through optimized hyperparameter tuning and sparse banded LD matrix approximation.

#### PGS-GS framework

**Figure 1:**
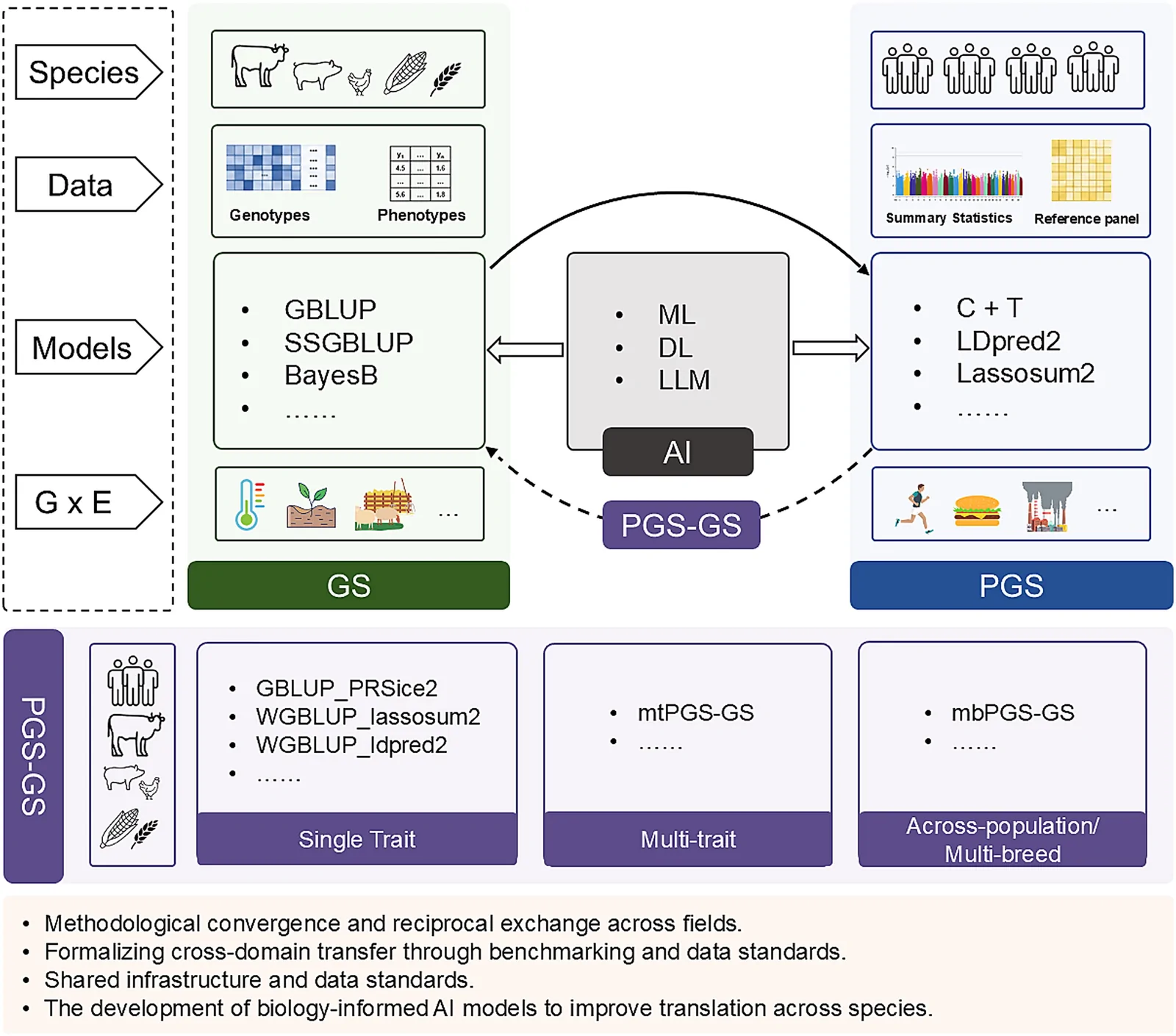
Conceptual overview of the PGS-GS framework. Bidirectional methodological exchange between human PGS and animal GS. The proposed PGS–GS framework facilitates the integration of human PGS methodologies into animal genomic prediction and provides a foundation for future multi-trait, multi-breed, and cross-population applications. GS: genomic selection, PGS: polygenic scores, G x E: genotype-by-environment interaction, GBLUP: genomic best linear unbiased prediction, SSGBLUP: single-step genomic best linear unbiased prediction, C + T: clumping and thresholding, AI: artificial intelligence, ML: machine learning, DL: deep learning, LLM: large language model.

The methodological foundations of human PGS and animal GS are highly interconnected. While statistical models originally developed for GS have substantially contributed to the advancement of PGS, methodological innovations in human genetics, particularly LD-aware modeling and summary-statistics-based Bayesian shrinkage methods, may in turn provide new opportunities for genomic prediction in agricultural species. Based on this mutual transferability, we propose a PGS–GS framework to facilitate methodological transfer from human genetics to animal genomic prediction (Fig. 1). Within this framework, the present study demonstrates a preliminary single-trait implementation integrating human PGS methodologies into animal GS. The PGS-GS is available on GitHub at https://github.com/jinbuwang/PGS-GS. Future studies will further extend these approaches to multi-trait, multi-breed, and cross-population genomic prediction in animal breeding.

(i) PGS-informed BLUP models

Three representative PGS methods were evaluated: C + T, LDpred2, and lassosum2. For PRSice2, SNPs retained under the optimal parameter settings were used to construct a reduced GRM, resulting in the GBLUP_PRSice2 model. For LDpred2 and lassosum2, posterior SNP effects were incorporated as marker-specific weights to construct weighted genomic relationship matrices (WGRMs). The WGRM implemented in HIBLUP was defined as:


$${G}_w=\frac{MW{M}^T}{\mathrm{tr}\left( MW{M}^T\right)/n}$$

where $M\in{\mathbb{R}}^{n\times m}$is the centered genotype matrix, $\mathrm{tr}\left( MW{M}^T\right)$ denotes the trace of $MW{M}^T$, and *n* is the number of individuals. *W* is a diagonal matrix containing marker-specific weights, defined as $W=\operatorname{diag}\left({w}_1,{w}_2,\dots, {w}_m\right)$. Marker weights were defined using posterior SNP effects estimated by LDpred2 or lassosum2: ${w}_j={\hat{\beta}}_j^2$. Posterior SNP effects estimated under LD-aware shrinkage models were used as marker-specific weights, thereby incorporating LD structure information into the weighting process. Using this strategy, two PGS-informed weighted BLUP models were established: WGBLUP_lassosum2 and WGBLUP_ldpred2.

(ii) PGS-informed kernel machine models

Posterior SNP effects derived from LDpred2 and lassosum2 were further used to construct weighted cosine similarity kernels. The weighted cosine kernel between individuals *i* and *j* was defined as:


$${K_{ij}}^{(w)}=\frac{\sum_{l=1}^m\ {w}_l{x}_{il}{x}_{jl}}{\sqrt{\sum_{l=1}^m\ {w}_l{x}_{il}^2}\sqrt{\sum_{l=1}^m\ {w}_l{x}_{jl}^2}}$$

where ${w}_l$ denotes the posterior effect-derived marker weight, and ${x}_{il}$ denotes the genotype of individual *i* at SNP *l*. The resulting kernels were subsequently incorporated into KRR and support vector regression (SVR), generating four nonlinear prediction models: KcRR_lassosum2, KcRR_ldpred2, ScVR_lassosum2, and ScVR_ldpred2.

(iii) Hybrid BLUP-kernel models

To further integrate kernel similarity learning into the BLUP framework, the weighted cosine kernels derived from LDpred2 and lassosum2 were directly incorporated into the covariance structure of the mixed model: $u\sim N\left(0,{K}_{\mathrm{cos}}{\sigma}_g^2\right)$, where ${K}_{\mathrm{cos}}$ denotes the weighted cosine similarity matrix. This strategy generated two hybrid prediction models: cos-GBLUP_lassosum2 and cos-GBLUP_ldpred2.

#### Compared methods

To evaluate the performance of PGS and PGS-GS approaches in animal breeding, we compared their prediction accuracy with six widely used GS and ML methods: GBLUP, BayesB, WGBLUP, GWABLUP, KRR, and SVR. Detailed descriptions of these methods are provided in the Supplementary Methods. KRR and SVR were implemented using the *scikit-learn* library in Python, with the ‘alpha’ and ‘C’ hyperparameters were selected from the grid {1 × 10^−5^, 1 × 10^−4^, 1 × 10^−3^, 1 × 10^−2^, 1 × 10^−1^, 1, 10, 20, 30, 40, 50}. GBLUP and WGBLUP were performed using the HIBLUP software [32]. BayesB was implemented in the *BGLR* package [33], with the ‘nIter’ and ‘burnIn’ parameters set to 50 000 and 10 000, respectively. GWAS was performed using the GCTA software [34]. In PRSice2, LD clumping windows were set to 50 kb for beef cattle and sheep, 300 kb for chickens, and 500 kb for pigs according to species-specific LD decay profiles (Fig. S1), with an LD ${r}^2$ threshold of 0.2. *P*-value thresholds ranged from 5 × 10^−8^ to 0.5 with increments of 5 × 10^−5^. Lassosum2 and LDpred2 were executed using the *bigsnpr* package [35]. LDpred2 was executed using the snp_ldpred2_auto function, with 30 logarithmically spaced initial values for the proportion of causal variants (*p*) ranging from 1 × 10^−4^ to 0.2. For lassosum2, four shrinkage parameters (*0.001,0.01,0.1,1*) and 30 regularization parameters were evaluated. To construct the LD reference panels required by these PGS algorithms, 1000 individuals were randomly sampled from the beef cattle and pig datasets, whereas all available individuals were utilized for the chicken and sheep datasets. All computations were performed on the same research server (CentOS Linux 8, 112 CPUs @ 2.00 GHz, 512 GB RAM), with 20 threads used for each analysis.

#### Datasets

To comprehensively evaluate the performance of PGS methods in animal breeding, we assembled four datasets comprising beef cattle, pigs, chickens, and sheep, encompassing a total of 20 traits (Table 1). Furthermore, to elucidate theoretical distinctions and practical implications, we compared the predictive performance of different models using SNP array and WGS data within beef cattle and pig. For four datasets, we conducted quality control (QC) for the imputed genotype data using PLINK 1.9 [36], excluding SNPs with a minor allele frequency ≤0.05, a Hardy–Weinberg equilibrium *P* ≤ 10^−6^, or a call rate <90%. For phenotypic data, missing values and outliers were removed. Phenotypes were pre-adjusted for fixed effects via linear regression to obtain phenotypic residuals, which were subsequently utilized for both genomic prediction and genome-wide association analyses.

**Table 1:** Summary of all datasets.

Datasets	Trait	*N* ^a^	Mean^b^	SD^b^	h^2^ ^c^
Cattle	LW	1405	519.12519.12	80.26	0.33
	DG_F	1410	0.95	0.19	0.30
	CW	1411	280.87280.87	51.77	0.40
	DP	1401	0.54	0.03	0.28
	NMW	1391	237.47237.47	44.64	0.35
	NMP	1387	0.45	0.03	0.06
	EMA	1401	86.3186.31	14.62	0.41
	HW	1407	24.5124.51	3.93	0.38
	CHW	1405	40.6640.66	6.67	0.50
Pig	BF	2771	10.9010.90	2.02	0.28
	LMD	2789	46.1546.15	3.82	0.35
	LMP	2782	54.0354.03	1.54	0.33
	TTN	2766	10.6910.69	1.01	0.29
Chicken	FEW	1052	42.4442.44	5.06	0.26
	EW36	1061	59.33	3.26	0.38
	EW56	1016	61.0461.04	4.23	0.26
	EW80	847	62.3262.32	4.83	0.30
Sheep	AFL	459	502.84502.84	93.87	0.17
	TP	508	6.756.75	3.37	0.07
	MLS	510	0.86	0.18	0.44

^a^The number of individuals with phenotypes.

^b^Mean and standard deviation (SD) of the phenotypic values.

^c^Heritability of traits. LW: live weight, DG_F: daily gain of fattening, CW: carcass weight, DP: dressing percentage, NMW: net meat weight, NMP: net meat percentage, EMA: eye muscle area, HW: head weight, CHW: cattle hide weight, BF: backfat thickness at 100 kg, LMD: loin muscle depth at 100 kg, LMP: lean meat percentage at 100 kg, TTN: total teat number, FEW: First egg weight. EW36: egg weight for 36 weeks, EW56: Egg weight for 56 weeks, EW80: Egg weight for 80 weeks, AFL: age at first lambing, TP: total prolificacy, MLS: maternal lamb survival.

#### Beef cattle dataset

The beef cattle dataset comprised 1421 Huaxi cattle, with detailed genotypic and phenotypic data sources described previously [37]. All individuals were genotyped using the Illumina BovineHD BeadChip that contained 770 000 SNPs. Following QC, 586 234 SNPs were retained. The array data were subsequently imputed to the WGS level using Beagle 5.5 [38], yielding a final set of 11 681 800 SNPs [39]. Nine traits with varying heritabilities were evaluated. Phenotypes were pre-adjusted for birth year, sex, farm, entry weight, and fattening time.

#### Pig dataset

The pig dataset included 2797 Duroc boars. These animals were genotyped with low-coverage WGS at a mean depth of 0.73× and imputed to high-coverage sequence data. After QC, 9 914 885 SNPs were remained for the subsequent analysis. To generate the corresponding array data, we updated the genomic coordinates of the GeneSeek Porcine 50 K array to the *Sus scrofa* 11.1 reference genome and extracted these specific loci from the WGS data, resulting in 31 155 SNPs. Phenotypes were pre-adjusted for fixed effects as detailed by Yang *et al.* [40].

#### Chicken dataset

The chicken dataset consisted of 1078 Rhode Island Red chickens genotyped using the Affymetrix 600 K chicken SNP [41]. Following rigorous QC, 1063 individuals and 294 705 SNPs were retained. We analyzed four phenotypes representing the entire egg-laying cycle: first egg weight (FEW) and egg weights at early (36 weeks), 56 weeks, and late (80 weeks) laying stages.

#### Sheep dataset

The sheep dataset comprised 538 Chios sheep from farms in northern Greece, genotyped using the Illumina Ovine SNP50K BeadChip [42]. After preprocessing and QC identically to the other datasets, the final dataset contained 520 individuals and 41 902 SNPs.

#### Evaluating genomic prediction performance

Predictive performance was evaluated using a five-fold cross-validation strategy. To ensure strict comparability, identical data partitions were applied across all models. A nested five-fold CV framework was used for all methods requiring parameter tuning, including ML models, PRSice2, and lassosum2: the outer five-fold CV was used for performance evaluation, while an inner five-fold CV within each training fold was used for hyperparameter optimization. The optimal hyperparameters identified in the inner loop were then used to retrain the model on the full outer training set prior to evaluation on the corresponding outer test set. Prediction accuracy was measured as the Pearson correlation coefficient between the pre-adjusted phenotypes and the genomic estimated breeding values (GEBV).

## Results

### Comparison of predictive accuracy of PGS methods across four datasets

**Figure 2:**
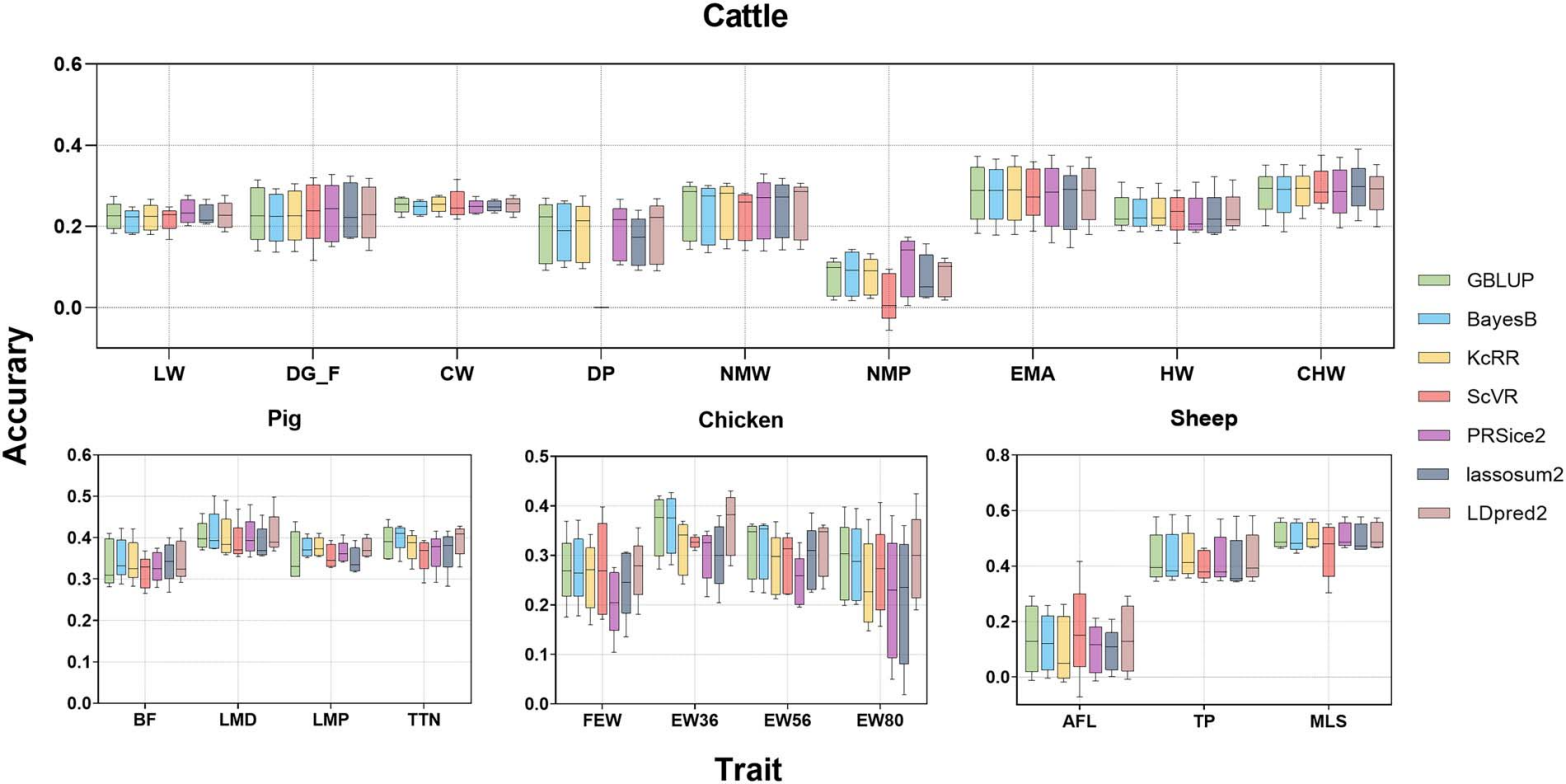
Predictive accuracy of GBLUP, BayesB, KcRR, ScVR, PRSice2, lassosum2, and LDpred2 based on SNP array data across four datasets.

We first evaluated the predictive performance of the three PGS algorithms across the animal datasets. Figure 2 illustrates the prediction accuracies of GBLUP, BayesB, KcRR, ScVR, PRSice2, lassosum2, and LDpred2 across the four animal datasets. The results demonstrate that, among the three PGS algorithms, LDpred2 exhibited the most consistent predictive accuracy, remaining comparable to or even superior to GBLUP overall. Specifically, in the pig and chicken datasets, LDpred2 generally achieved the highest or second-highest prediction accuracy among all algorithms, improving accuracy over GBLUP by 6% for LMP and by 3% for EW80. In the beef cattle and sheep datasets, the predictive accuracy of LDpred2 was almost identical to that of GBLUP. Interestingly, PRSice2 was also highly competitive in the beef cattle dataset, improving accuracy over GBLUP by 5% and 38% for the LW and NMP traits, respectively. lassosum2 showed the poorest performance across the four datasets. Notably, the predictive accuracy of the LDpred2 model was highly consistent with that of BayesB. Furthermore, while the two ML algorithms outperformed GBLUP for certain traits, they did not demonstrate a systematic advantage over the linear models.

### Comparison of predictive accuracy of different weighted GBLUP models across four datasets

**Figure 3:**
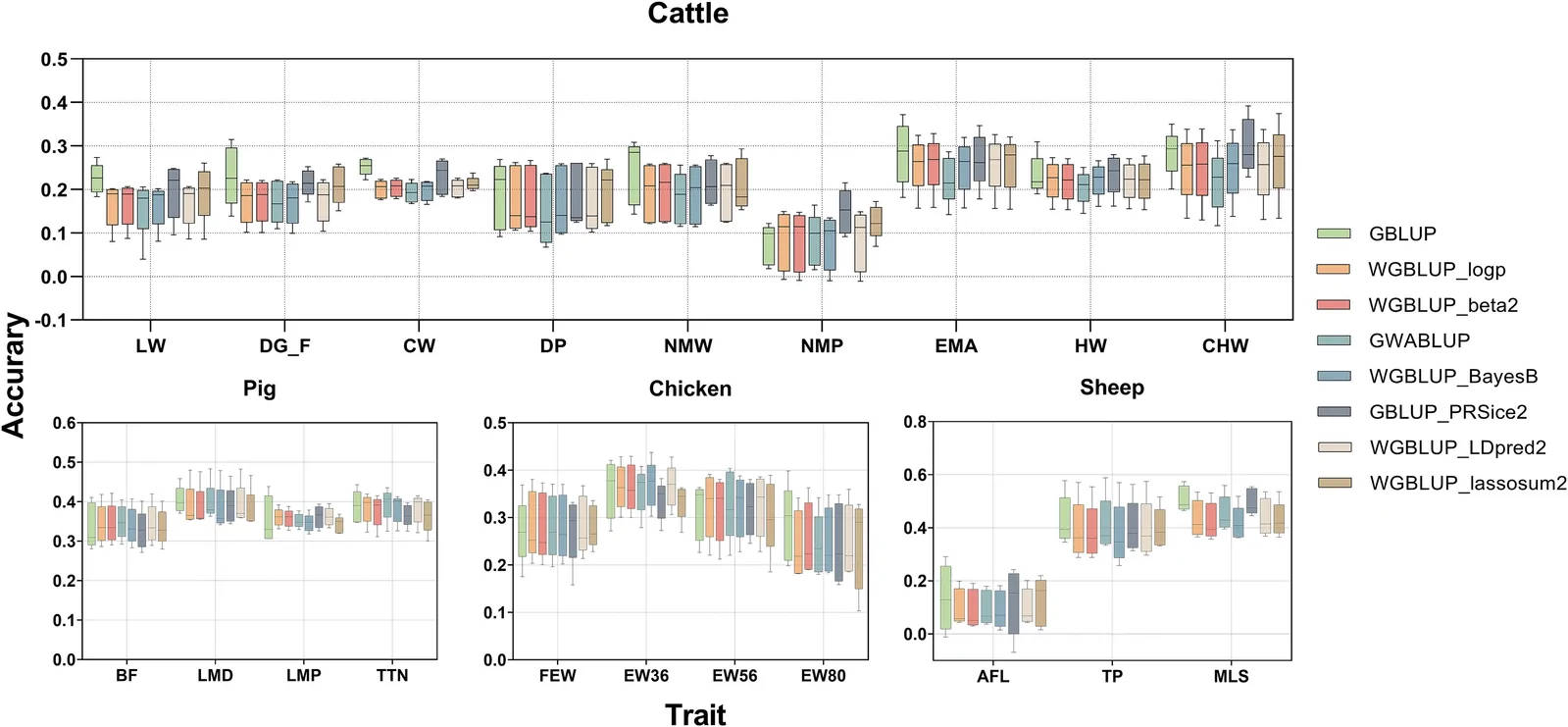
Comparison of predictive accuracy of GBLUP, WGBLUP_logp, WGBLUP_beta2, GWABLUP, WGBLUP_BayesB, WGBLUP_PRSice2, WGBLUP_LDpred2, and WGBLUP_lassosum2 based on SNP array data across four datasets.

By integrating the three PGS algorithms with traditional GBLUP, we developed three novel PGS-GS models: GBLUP_PRSice2, WGBLUP_LDpred2, and WGBLUP_lassosum2. Here, we compared the performance of seven algorithms constructed based on GWAS summary statistics or WGRMs against the conventional GBLUP model. Figure 3 illustrates the prediction accuracies of the eight algorithms using array data across the four datasets. The results indicated that in the beef cattle and sheep datasets, the prediction accuracies of the WGBLUP models were generally lower than that of standard GBLUP. Notably, the three PGS-GS algorithms exhibited better performance than the other WGBLUP models. Among these, the prediction accuracies of GBLUP_PRSice2 and WGBLUP_lassosum2 were generally higher than that of WGBLUP_LDpred2. For the AFL trait, WGBLUP_lassosum2 yielded an average improvement of 38% over the four traditional weighted GBLUP models (WGBLUP_logp, WGBLUP_beta2, GWABLUP, and WGBLUP_BayesB). In the pig and chicken datasets, various weighted GBLUP models frequently displayed higher prediction accuracies than GBLUP for certain traits. Additionally, the predictive performance of WGBLUP_LDpred2 outperformed both GBLUP_PRSice2 and WGBLUP_lassosum2. In the pig dataset, WGBLUP_LDpred2 improved prediction accuracy by 5% for the backfat thickness at 100 kg (BF) trait compared to GBLUP, while GBLUP_PRSice2 improved by 2% for the LMP trait. In the chicken dataset, excluding the EW80 trait, WGBLUP_LDpred2 increased prediction accuracy by an average of 3% across the remaining three traits relative to GBLUP. GWABLUP also demonstrated superior predictive ability, achieving the highest prediction accuracies for traits such as BF and EW56, with an average improvement of 4% over GBLUP. WGBLUP_logp and WGBLUP_beta2 exhibited comparable predictive capacities across the four datasets, whereas the prediction accuracy of WGBLUP_BayesB was generally lower than that of WGBLUP_LDpred2.

### Comparison of predictive accuracy of 20 models across four datasets

**Figure 4:**
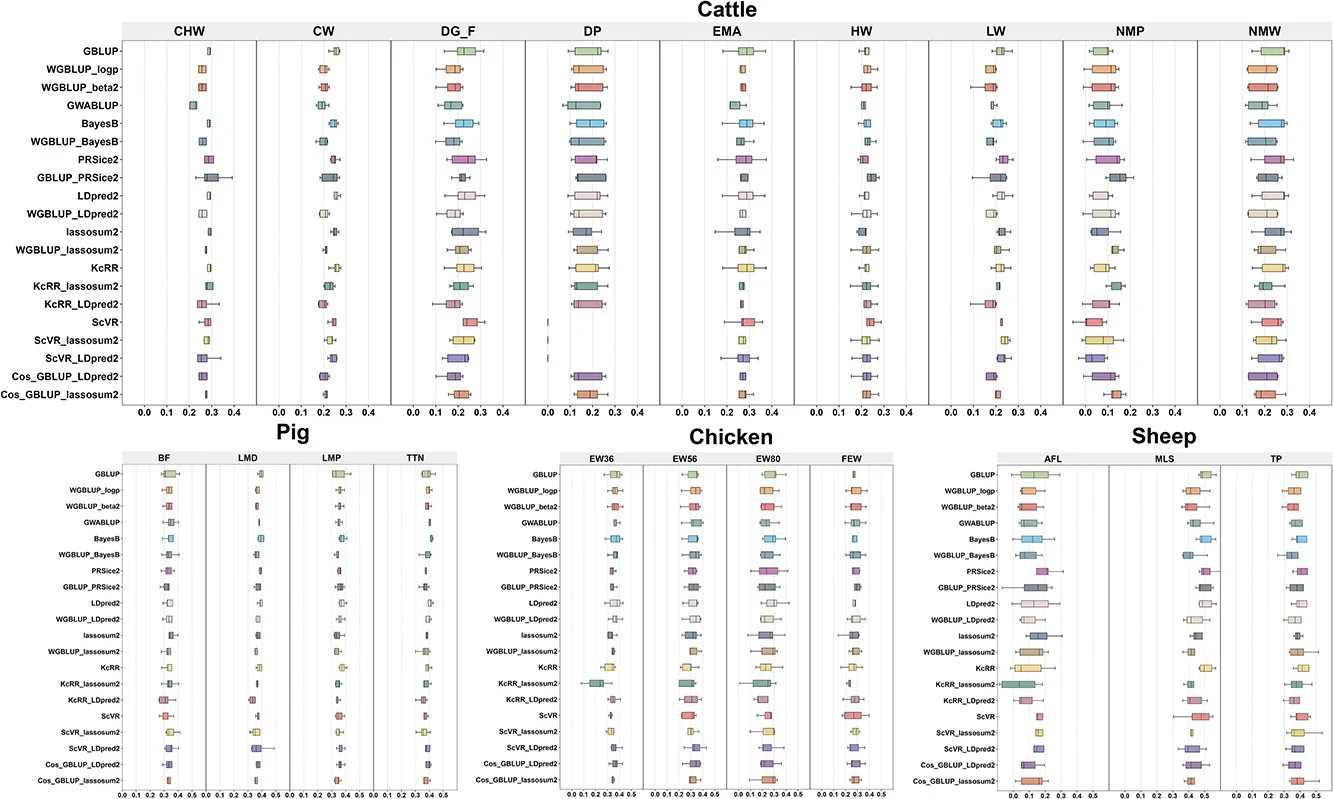
Predictive accuracy of 20 models based on SNP array data across four datasets.

To comprehensively evaluate our proposed PGS-GS framework, we assessed the prediction accuracies of 20 models using array data across the four datasets. These included GBLUP, BayesB, GWABLUP, three WGBLUP models, three PGS algorithms, two ML algorithms, and nine PGS-GS models (Fig. 4). The results demonstrate that LDpred2 provided the most consistent predictive accuracy across the four datasets, and the PGS-GS models were competitive. For example, ScVR_lassosum2 obtained the highest accuracy for the BF trait, yielding a 4% improvement over GBLUP. Furthermore, ScVR_lassosum2 and ScVR_LDpred2 achieved the highest prediction accuracies for the FEW and EW56 traits, outperforming GBLUP by 5% and 8%, respectively. Regarding the three BLUP-based PGS-GS models, their prediction accuracies were often lower than those of their baseline models. For instance, in the beef cattle dataset, the prediction accuracy of WGBLUP_lassosum2 was lower than that of lassosum2 across all traits. However, in the chicken dataset, the PGS-GS models generally outperformed their baseline models (excluding the EW80 trait); for example, WGBLUP_LDpred2 improved the average prediction accuracy across the remaining three traits by 3% compared to LDpred2. For the four ML-based extended models, we also observed that their prediction accuracies were lower than those of the independent LDpred2 and lassosum2 algorithms in the beef cattle and sheep datasets. Conversely, in the pig and chicken datasets, ScVR_lassosum2 improved prediction accuracy by 10% for the BF trait compared to ScVR, while ScVR_LDpred2 improved accuracy by 19% for the EW56 trait relative to ScVR. Finally, for the two extended models combining BLUP with the kernel trick, their prediction accuracies were largely comparable to those of their corresponding baseline models.

### Comparison of predictive accuracy of different models based on SNP array and sequencing data in beef cattle and pigs

**Figure 5:**
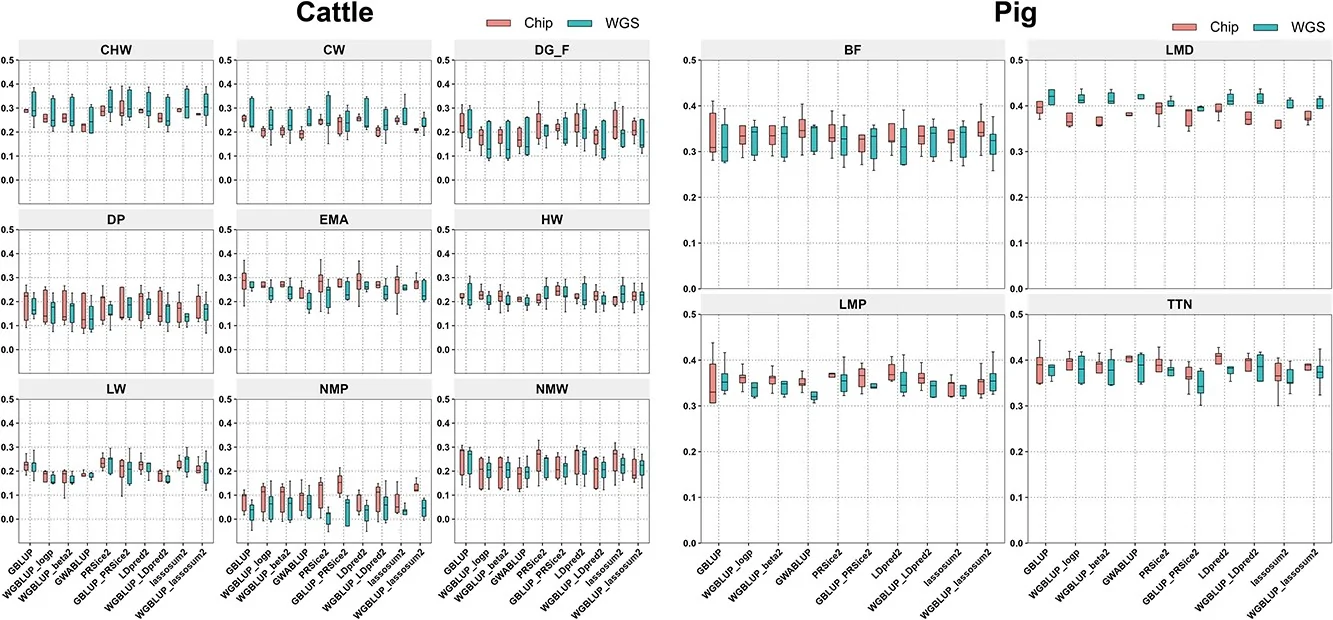
Predictive accuracy of different models based on SNP array and sequencing data in cattle and pig.

To evaluate the generalization capabilities of the PGS and PGS-GS algorithms on WGS data and to investigate the impact of data dimensionality on predictive performance, we benchmarked 10 linear prediction models in this section. Given the prohibitive computational complexity and time costs associated with applying ML and the BayesB model to WGS data, they were excluded from this evaluation. Figure 5 illustrates the prediction accuracies of these 10 models using array and WGS data in the beef cattle and pig datasets. Overall, compared to conventional array data, the WGS data did not yield systematic improvements in predictive performance. Specifically, in the beef cattle dataset, WGS data demonstrated a significant advantage solely for the cattle hide weight (CHW) and carcass weight (CW) traits, the GBLUP model utilizing WGS data improved prediction accuracies by 8% and 6% for these two traits, respectively, compared to array data. For the live weight (LW) trait, the predictive performance of all models remained largely comparable or showed only marginal gains across both data dimensions. Similarly, in the pig dataset, WGS data achieved higher prediction accuracies than array data exclusively for the loin muscle depth at 100 kg (LMD) trait. When evaluating the performance of individual algorithms driven solely by WGS data, LDpred2 maintained robust predictive accuracy in the beef cattle dataset. However, in the pig dataset, no single algorithm emerged as the optimal performer across all traits. Taken together, when processing high-dimensional WGS data, GBLUP remained the most robust algorithm, whereas the predictive accuracies of the various WGBLUP extensions were generally inferior to that of GBLUP.

### Distribution of SNP weights

**Figure 6:**
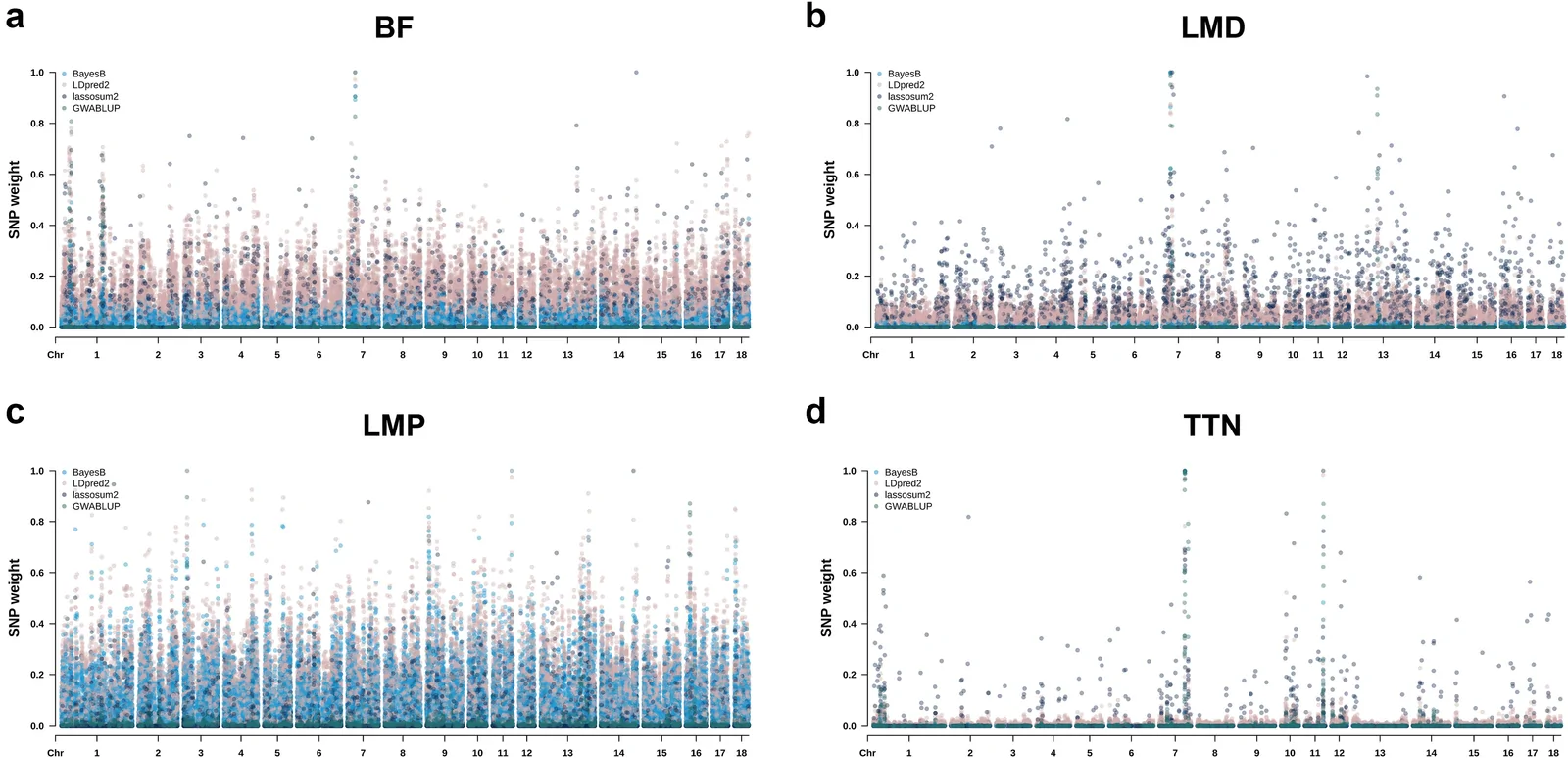
Distribution of SNP weights derived from BayesB, LDpred2, lassosum2, and GWABLUP in the pig dataset. Manhattan plots of SNP weights for BF (A), LMD (B), LMP (C), and TTN (D).

To further elucidate the underlying mechanisms of the PGS models and their extended algorithms within animal breeding, we visualized the distribution of SNP effects estimated by the BayesB, LDpred2, lassosum2, and GWABLUP models across the four species datasets. Figure 6 illustrates the absolute SNP effect distribution profiles of these four models in the pig dataset, and results for the beef cattle, sheep, and chicken datasets are provided in the Figs S2–S4. Our results indicate that BayesB and LDpred2 exhibited highly consistent shrinkage profiles in their effect distributions, providing a direct explanation for their comparable prediction accuracies. Both algorithms effectively captured QTL significantly associated with the traits, forming distinct “effect peaks” on the Manhattan plots, while simultaneously shrinking the effects of the vast polygenic background loci to infinitesimally small, non-zero values. However, micro-level differences in local weight allocation were observed between the two methods: BayesB tended to emphasize a few core SNPs with massive effects, whereas LDpred2 demonstrated a smoother shrinkage pattern that retained a greater number of collinear loci. Benefiting from this capacity to accommodate both major QTL and the diffuse polygenic background, the prediction accuracies of BayesB and LDpred2 were comparable to that of the standard GBLUP model, which strictly relies on the infinitesimal assumption. Furthermore, extracting the posterior variances from these two algorithms to construct weighted GBLUP models precisely assigned higher genetic weights to the significant QTL, thereby further enhancing the predictive performance of the extended models. In contrast, the lassosum2 algorithm exhibited extreme sparsity. This algorithm directly compressed the effects of the vast majority of SNPs to zero, leaving only a limited number of non-zero loci discretely scattered across the genome. When constructing its corresponding WGBLUP model, even if an infinitesimally small non-zero constant was artificially assigned to these zero-effect loci, the variance contribution of the weak polygenic background suffered a magnitude collapse during the squared weighting calculation of the GRM. Consequently, this background signal was completely overshadowed by the few large-effect loci, ultimately leading to a deterioration rather than an improvement in the prediction accuracy of the lassosum2-derived WGBLUP model. Additionally, we observed that for traits driven by strong major genes (the total teat number (TTN), which is controlled by a major QTL on chromosome 7), GWABLUP demonstrated an effect-capturing capability highly analogous to that of LDpred2. By assigning elevated variance weights to these major-effect regions, GWABLUP achieved a prediction accuracy superior to that of traditional GBLUP.

Finally, we evaluated the computational efficiency of the various algorithms across different data dimensions. The results demonstrated that GBLUP exhibited the shortest single-run execution time among all evaluated algorithms. BayesB had the longest execution time; in the beef cattle dataset, the average runtime for a single trait reached up to 1370 minutes (Table S2). Furthermore, the PGS algorithms displayed remarkable computational efficiency. Specifically, the average runtime for PRSice2 was merely 1–2 minutes. For LDpred2 and lassosum2, provided that the LD matrices were pre-constructed, their execution efficiencies were also exceedingly high. Notably, although both employ a Bayesian framework, the computational speed of LDpred2 was over a hundred times faster than that of BayesB, and the overall efficiency of lassosum2 was even superior to that of LDpred2. When applied to WGS data, the computational advantages of the PGS algorithms were further amplified, with total execution times remaining stably controlled at ~1 hour (Table S1). Additionally, the ML algorithms were computationally slower than the GBLUP and PGS approaches, but significantly faster than BayesB. The computational efficiency of KRR was markedly superior to that of SVR. However, due to the non-linear growth in the computational complexity of kernel matrices, these ML algorithms will encounter severe memory and time bottlenecks when processing WGS data.

## Discussion

Genomic research in human genetics and animal breeding shares a highly homologous methodological foundation. However, fundamental differences in research objectives, population genetic architectures, and phenotypic systems dictate distinct analytical paradigms, sample size requirements, and application scenarios [43]. This disciplinary separation has resulted in deep but compartmentalized expertise. In human genetics, numerous studies improved the accuracy of PGS by leveraging methodologies originally developed for GS [9, 44, 45]. Conversely, the transfer of human genetic methodologies to animal breeding remains comparatively underexplored, particularly in the context of integrating LD-aware PGS approaches into genomic prediction frameworks.

Numerous studies have demonstrated that the performance of genomic prediction models largely reflects the underlying genetic architecture of the trait in question [11]. Bayesian methods generally perform better for traits dominated by a few major-effect QTLs, whereas BLUP methods are more suitable for highly polygenic traits with predominantly small-effect loci. However, our results indicate that evaluating model performance solely based on the genetic architecture of a trait is incomplete. We posit that the optimal prediction model is jointly determined by the underlying logic of the algorithm, the macroscopic LD profile of the species, and the microscopic genetic architecture of the trait. Wray *et al.* noted that effective population size (Ne) is a key factor driving the differences in analytical frameworks and predictive performance between livestock GEBV and human PGS [46]. Our findings are consistent with this observation. Commercial pigs and chickens, which have undergone intensive directional selection within closed nucleus populations, generally exhibit relatively small historical effective population sizes. These populations are associated with slower LD decay compared with cattle and sheep in this study. Under these conditions, LDpred2 and BayesB showed relatively strong and consistent predictive performance across multiple traits. This may reflect their ability to model correlated markers under LD-aware or Bayesian shrinkage frameworks, which can better utilize persistent LD information across genomic regions [29]. In contrast, the beef cattle dataset exhibited the fastest LD decay among the studied populations, while the sheep dataset was characterized by a relatively small sample size and higher phenotypic noise. These factors may reduce the stability of LD estimation and increase uncertainty in marker effect estimation. Accordingly, PRSice2 showed competitive performance in the beef cattle dataset, which may be attributed to its pruning strategy that reduces the influence of noisy variants and improves predictive stability under weaker LD signal conditions. Nevertheless, substantial heterogeneity in predictive performance was observed across traits and populations, indicating that no single algorithm consistently outperformed others under all genetic architectures and population structures.

Studies on weighting strategies suggest that while weighting enables models to adapt to specific genetic architectures, the intrinsic quality of the weights is the critical determinant of success in genomic prediction [22, 23, 47]. Our findings similarly demonstrate that traditional weighted GBLUP strategies, if directly reliant on GWAS results with substantial estimation errors, paradoxically amplify model noise and compromise prediction accuracy. The PGS-GS framework proposed herein not only jointly models marker effects but also adequately accounts for local LD, thereby extracting more robust prior weights than conventional GWAS-based weighting. Furthermore, this study confirms that extending this prior information to non-linear ML models holds potential for the prediction of complex traits in livestock and poultry. However, it is crucial to note that, constrained by double shrinkage, the predictive upper bound of weighted models rarely exceeds that of the source algorithm used to derive the weights [22]. Concurrently, WGS data has yet to fully realize its potential in animal genomic prediction, likely due to the imbalance between the extremely high dimensionality of sequence variants and currently limited sample sizes. In particular, the inclusion of large numbers of low-frequency and weak-effect variants may increase estimation noise and reduce signal-to-noise ratios under current sample sizes. [48–50]. Moreover, commercial SNP arrays are already enriched for common variants tagging major haplotypes, such that a substantial proportion of additive genetic variance may already be captured by array markers, thereby limiting the incremental predictive value of WGS data under current conditions. Finally, from a practical standpoint, bolstered by the mature theoretical frameworks and highly optimized software ecosystems of human genetics, PGS algorithms exhibit undeniably superior performance in both computational and memory efficiency.

The application of human-derived PGS algorithms to agricultural species inherently necessitates a reliance on GWAS summary statistics. In human genetics, bolstered by large-scale public consortia and massive cohorts, the estimation precision of GWAS effects is exceptionally high [51, 52]. By contrast, the livestock sector is increasingly constrained by data privacy concerns and commercial barriers, rendering the large-scale aggregation of raw genotypic and phenotypic data highly impractical [1]. Consequently, the summary statistics-based PGS frameworks established in human genetics, which circumvent the need to share sensitive raw data, perfectly align with the imperative to dismantle agricultural “data silos.” This paradigm is poised to become a pivotal trajectory for the future of animal breeding. Conversely, the mature genotype-by-environment interaction models developed in plant and animal breeding can offer critical cross-disciplinary insights to enhance the predictive accuracy of human PGS [43]. Nevertheless, the current study represents only a preliminary single-trait implementation of the proposed PGS–GS framework, and its generalizability across multi-trait, multi-breed, and cross-population settings remains to be systematically evaluated. In addition, only three representative PGS methods were investigated in this study, and the integration potential of other emerging human genetic methodologies warrants further exploration. In future studies, we aim to further extend this framework to multi-trait joint analyses, as well as to the highly challenging domain of multi-breed and cross-population prediction in both humans and livestock [53, 54]. Therefore, we advocate for deep methodological convergence and reciprocal exchange between human genetics and animal breeding. By establishing shared computational infrastructures and unified data standards, the profound integration of these two disciplines will likely accelerate the development of next-generation genomic prediction frameworks.

## Conclusion

In this study, we proposed a PGS–GS framework to facilitate the transfer of human PGS methodologies into animal genomic prediction and demonstrated a preliminary single-trait implementation based on weighting strategies. Our results suggest that the optimal prediction model is determined not only by the algorithm and the trait’s genetic architecture, but also by the species-level LD profile. LDpred2 achieved predictive accuracy comparable to or even better than GBLUP, and among the weighted models, the PGS-GS models outperformed conventional weighted GBLUP. We also emphasize that the full potential of WGS data in animal breeding has yet to be realized. Therefore, the greater value of applying human PGS methods to animal GS lies in their computational efficiency and summary-statistics-based, data-sharing-compatible design. Looking forward, multi-trait and multi-breed predictions remain highly active research frontiers in both disciplines. Overall, this study provides a comparative framework for algorithm selection across different population genomic contexts and emphasizes the value of methodological integration between human genetics and animal breeding for advancing next-generation genomic prediction.

**Key Points**

- We integrated human-derived polygenic scores (PGS) methodologies into animal genomic prediction.
- We developed a PGS–GS framework based on weighting strategies and evaluated its performance in linear and nonlinear models.
- We compared SNP array and whole-genome sequencing (WGS) data, highlighting the role of linkage disequilibrium (LD) in genomic prediction.

## Supplementary Material

Supplementary_materials_bbag397

## Data Availability

Beef cattle dataset: The GWAS summary statistics for the Huaxi cattle dataset are available at https://figshare.com/articles/dataset/32261241, The raw genotype and phenotype data are available from the corresponding authors on reasonable request, Pig dataset: https://gigadb.org/dataset/100894, Chicken Dataset: https://figshare.com/articles/dataset/Genome-wide_Association_Analysis_of_Age-Dependent_Egg_Weights_in_Chickens/5844420, Sheep Dataset: https://data.mendeley.com/datasets/nvjn8mvwtg/1.

## Notes

Jinbu Wang focuses on the development of genomic selection algorithms, and Lili Du focuses on mechanistic insights from multi-omics integrative analysis.
