# Supplementary material for: PGS-GS: a framework integrating polygenic scores and genomic selection in animal breeding
Source: Brief Bioinform. 2026 Sep 1;27(5):bbag397. doi: 10.1093/bib/bbag397 (PMC13533199; doi:10.1093/bib/bbag397)
Supplement: Supplementary file 1 [file supplementary_materials_bbag397.docx]

**Supplement Methods**

**Linear methods**

**GBLUP**

GBLUP is one of the most widely used genomic prediction models in animal breeding [1], as follows:

$$y=1\mu+Zg+e,$$

where y is the phenotype vector. μ is the overall mean, and $g$ and $e$ are the vectors of genomic breeding values and residuals, assumed to follow $g\sim N\left( 0,G\sigma_{g}^{2} \right)$and $e\sim N\left( 0,I\sigma_{e}^{2} \right)$, respectively. Z is an incidence matrix linking $g$ to y. The genomic relationship matrix G implemented in HIBLUP was calculated as:

$$G=\frac{{MM}^{T}}{\mathrm{tr}\left( {MM}^{T} \right)/n}$$

where $M\in\mathbb{R}^{n\times m}$is the centered genotype matrix, $\mathrm{tr}\left( {MM}^{T} \right)$ denotes the trace of ${MM}^{T}$ $n$ is the number of individuals, and $m$ is the number of SNP markers.

**BayesB**

BayesB assumes that most SNPs have no effect, whereas a small proportion of SNPs have non-zero effects with marker-specific variances [2]. The BayesB model can be expressed as:

$$\mathbf{y}=\mathbf{1}\mu+\sum_{j=1}^{m} \mathbf{x}_{j}\beta_{j}+\mathbf{e}$$

where $\mathbf{y}$**,** $\mu$ and $\mathbf{e}$ are defined as above, $\mathbf{x}_{j}$ is the genotype vector of SNP $j$ across individuals; $\beta_{j}$ is the additive effect of the $j$-th marker. In BayesB, marker effects are assumed to follow a mixture prior:

$\beta_{j}=\left\{ \begin{aligned} &0,&& \text{with probability}\text{ }\pi\\ &N(0,\sigma_{\beta_{j}}^{2}), && \text{with probability}\text{ }1-\pi&& \end{aligned} \right.$,

where π is the prior proportion of markers with zero effect. The marker-specific variance is assumed to follow a scaled inverse chi-square distribution: $\sigma_{\beta_{j}}^{2}～\chi^{-2}\left( \nu,S \right)$, where $\nu$and $S$denote the degrees of freedom and scale parameter, respectively.

**WGBLUP**

WGBLUP extends the standard GBLUP framework by assigning marker-specific weights to SNPs to account for heterogeneous marker variances [3]. In this study, three commonly used weighting strategies were implemented:

(1) **WGBLUP_logp**: $w_{j}=-log_{10}(P_{j})$

(2) **WGBLUP_beta2**: $w_{j}=\hat{\beta}_{GWAS,j}^{2}$

(3) **WGBLUP_BayesB**: $w_{j}=\hat{\beta}_{BayesB,j}^{2}$

**GWABLUP**

GWABLUP uses GWAS results to assign differential weights to all SNPs. Specifically, it is calculated according to the method described by Theo Meuwissen et al. [4]:

$$PP_{j}=\frac{\pi e^{LR_{j}}}{\left[ \pi e^{LR_{j}}+(1-\pi) \right]}$$

where, $PP_{j}$ represents the weight of each SNP; $\pi$ is the prior probability that SNPs have a substantial effect. $LR_{j}$ denotes the moving average of the log-likelihood-ratio (LR) of five SNPs, and LR can be calculated by $1/2\left( \hat{b}/se \right)^{2}$, where $\hat{b}$ is the SNP effect estimate and $\mathrm{se}$ is its standard error.

**Machine learning methods**

**Kernel ridge regression**

KRR combines ridge regression with kernel methods, enabling the modeling of nonlinear relationships through linear regression in a reproducing kernel Hilbert space [5]. The KRR prediction model can be expressed as:

$$\hat{y}=k^{T}(K+\lambda I)^{-1}y$$

where K is the Gram matrix, $k$ is the kernel vector between the test sample and the training samples, $I$ is the identity matrix, and λ is the regularization parameter. In this study, KcRR [6] was used, which employs the cosine kernel defined as:

$$K_{ij}=\frac{\sum_{l=1}^{m} x_{il}x_{jl}}{\sqrt{\sum_{l=1}^{m} x_{il}^{2}}\sqrt{\sum_{l=1}^{m} x_{jl}^{2}}}$$

where $x_{i}$and $x_{j}$are the genotype vectors of individuals $i$and $j$, respectively.

**Support vector regression**

Support vector regression (SVR) maps input data into a high-dimensional feature space using a kernel function and fits a regression function under the $\varepsilon$-insensitive loss framework[7, 8]. In $\varepsilon$-SVR, errors within the margin $\varepsilon$are ignored, whereas errors exceeding $\varepsilon$are penalized. The dual form of the SVR prediction function is:

Where is the kernel function, $\hat{\alpha}_{i}$ and $\alpha_{i}$ are the Lagrange multipliers, *b* is the bias term. In this study, SVR was implemented using the cosine kernel.

1.	VanRaden PM. Efficient methods to compute genomic predictions, J Dairy Sci 2008;91:4414-4423.

2.	Meuwissen TH, Hayes BJ, Goddard ME. Prediction of total genetic value using genome-wide dense marker maps, Genetics 2001;157:1819-1829.

3.	Su G, Christensen OF, Janss L et al. Comparison of genomic predictions using genomic relationship matrices built with different weighting factors to account for locus-specific variances, Journal of Dairy Science 2014;97:6547-6559.

4.	Meuwissen T, Eikje LS, Gjuvsland AB. GWABLUP: genome-wide association assisted best linear unbiased prediction of genetic values, Genet Sel Evol 2024;56:17.

5.	Exterkate P, Groenen PJF, Heij C et al. Nonlinear forecasting with many predictors using kernel ridge regression, International Journal of Forecasting 2016;32:736-753.

6.	An B, Liang M, Chang T et al. KCRR: a nonlinear machine learning with a modified genomic similarity matrix improved the genomic prediction efficiency, Brief Bioinform 2021;22.

7.	Cortes C, Vapnik V. Support-vector networks, Machine Learning 1995;20:273-297.

8.	Wang J, Zong W, Shi L et al. Using mixed kernel support vector machine to improve the predictive accuracy of genome selection, Journal of Integrative Agriculture 2026;25:775-787.

 Figure S1 LD decay plots for the four datasets.

Figure S2 Distribution of SNP weights derived from BayesB, LDpred2, lassosum2, and GWABLUP in the cattle dataset. Manhattan plots of SNP weights for CHW (A), CW (B), DG_F (C), DP (D), EMA (E), HW (F), LW(G),NMP (H) and NMW (I).

Figure S3 Distribution of SNP weights derived from BayesB, LDpred2, lassosum2, and GWABLUP in the chicken dataset. Manhattan plots of SNP weights for EW36 (A), EW56 (B), EW80 (C), and FEW (D).

Figure S4 Distribution of SNP weights derived from BayesB, LDpred2, lassosum2, and GWABLUP in the sheep dataset. Manhattan plots of SNP weights for AFL (A), MLS (B) and TP (C).

Table S1 Runtime of different models based on sequencing data in cattle and pigs. (minutes)

|  | Traits | GBLUP | WGBLUP_  logp | WGBLUP_  beta2 | GWA  BLUP | PRSice2 | GBLUP_  PRSice2 | LDpred2 | WGBLUP_  LDpred2 | lassosum2 | WGBLUP_  lassosum2 |
| --- | --- | --- | --- | --- | --- | --- | --- | --- | --- | --- | --- |
| Cattle | LW | 2 | 6.27 | 5.31 | 15.34 | 25.89 | 25.89 | 106.81 | 8.8 | 50.6 | 8.28 |
|  | DG_F | 2 | 5.6 | 5.12 | 17.1 | 26.1 | 26.1 | 105.4 | 8.38 | 49.35 | 7.9 |
|  | CW | 2.02 | 5.61 | 5.03 | 16.21 | 25.83 | 25.83 | 103.11 | 8.16 | 51.55 | 7.99 |
|  | DP | 2.04 | 5.71 | 5.01 | 15.97 | 24.13 | 24.13 | 102.95 | 8.43 | 53.76 | 7.91 |
|  | NMW | 1.99 | 5.58 | 4.93 | 16.99 | 24.74 | 24.74 | 105.9 | 8.09 | 52.47 | 7.77 |
|  | NMP | 1.98 | 5.54 | 4.97 | 16.3 | 23.16 | 23.16 | 107.93 | 8.44 | 53.76 | 7.99 |
|  | EMA | 2.02 | 5.68 | 4.98 | 17.18 | 22.93 | 22.93 | 107.08 | 8.14 | 54.11 | 8.01 |
|  | HW | 2.01 | 5.51 | 5 | 17.11 | 25.34 | 25.34 | 108.3 | 8.3 | 53.08 | 7.79 |
|  | CHW | 1.98 | 5.52 | 5.03 | 17.01 | 26.72 | 26.72 | 104.1 | 8.04 | 53.8 | 7.98 |
| Pig | BF | 4.41 | 7.38 | 7.57 | 12.82 | 23.79 | 23.79 | 97.29 | 53.37 | 42.91 | 55.51 |
|  | LMD | 4.54 | 7.43 | 7.47 | 12.53 | 24.69 | 24.69 | 99.6 | 54.24 | 41.39 | 56.08 |
|  | LMP | 4.63 | 7.61 | 7.26 | 12.8 | 22.47 | 22.47 | 91.34 | 54.31 | 40.38 | 56.51 |
|  | TTN | 4.43 | 7.41 | 7.19 | 13.59 | 23.48 | 23.48 | 91.28 | 52.88 | 39.15 | 55.22 |

|  | Traits | GBLUP | WGBLUP_  logp | WGBLUP_beta2 | GWABLUP | BayesB | WGBLUP_BayesB | PRSice2 | GBLUP_PRSice2 | LDpred2 | WGBLUP_LDpred2 | lassosum2 | WGBLUP_lassosum2 | KcRR | KcRR_  lassosum2 | KcRR_  LDpred2 | ScVR | ScVR_  lassosum2 | ScVR_  LDpred2 | Cos_  GBLUP_LDpred2 | Cos_  GBLUP_  lassosum2 |
| --- | --- | --- | --- | --- | --- | --- | --- | --- | --- | --- | --- | --- | --- | --- | --- | --- | --- | --- | --- | --- | --- |
| Cattle | LW | 0.12 | 0.3 | 0.29 | 0.52 | 1425.07 | 0.71 | 2.1 | 2.22 | 118.25 | 1.24 | 65.95 | 1.21 | 1.31 | 2.87 | 2.77 | 1.09 | 2.84 | 2.65 | 0.16 | 0.16 |
|  | DP | 0.12 | 0.28 | 0.27 | 0.47 | 1472.1 | 0.68 | 2.14 | 2.26 | 120.02 | 1.2 | 68.19 | 1.18 | 1.18 | 2.75 | 2.86 | 1.05 | 2.77 | 2.6 | 0.15 | 0.16 |
|  | NMW | 0.12 | 0.29 | 0.27 | 0.47 | 1356.02 | 0.7 | 2.16 | 2.28 | 128.6 | 1.22 | 65.47 | 1.19 | 1.32 | 2.77 | 2.66 | 1.12 | 2.61 | 2.71 | 0.15 | 0.16 |
|  | NMP | 0.12 | 0.28 | 0.27 | 0.47 | 1298.69 | 0.62 | 2.14 | 2.26 | 116.88 | 1.21 | 70.64 | 1.17 | 1.37 | 2.73 | 2.69 | 1.02 | 2.59 | 2.75 | 0.14 | 0.15 |
|  | EMA | 0.12 | 0.29 | 0.28 | 0.47 | 1500.3 | 0.67 | 2.17 | 2.29 | 121.05 | 1.19 | 70.56 | 1.19 | 1.33 | 2.78 | 2.68 | 1.06 | 2.84 | 2.87 | 0.18 | 0.17 |
|  | HW | 0.12 | 0.28 | 0.27 | 0.47 | 1372.53 | 0.68 | 2.13 | 2.25 | 118.79 | 1.25 | 65.33 | 1.24 | 1.31 | 2.85 | 2.74 | 1.09 | 2.7 | 2.92 | 0.16 | 0.18 |
|  | CW | 0.13 | 0.29 | 0.28 | 0.47 | 1265.21 | 0.7 | 2.17 | 2.3 | 113.16 | 1.21 | 66.67 | 1.17 | 1.19 | 2.71 | 2.7 | 1.12 | 2.88 | 2.96 | 0.15 | 0.15 |
|  | DG_F | 0.12 | 0.27 | 0.28 | 0.47 | 1334.71 | 0.71 | 2.14 | 2.26 | 110.22 | 1.14 | 66.15 | 1.11 | 1.16 | 2.8 | 2.71 | 1.04 | 3.07 | 2.8 | 0.14 | 0.17 |
|  | CHW | 0.12 | 0.28 | 0.28 | 0.47 | 1323.68 | 0.72 | 2.15 | 2.27 | 124.04 | 1.21 | 66.48 | 1.22 | 1.35 | 2.75 | 2.7 | 1.1 | 2.83 | 2.79 | 0.15 | 0.18 |
| Pig | BF | 0.08 | 0.1 | 19.94 | 0.12 | 256.91 | 0.14 | 0.94 | 1.02 | 0.99 | 0.64 | 1.27 | 0.68 | 6.48 | 10.61 | 14.08 | 6.62 | 10.55 | 14.15 | 0.78 | 0.68 |
|  | LMD | 0.08 | 0.11 | 16.58 | 0.11 | 257.27 | 0.14 | 0.94 | 1.02 | 0.78 | 0.67 | 1.23 | 0.65 | 6.54 | 10.57 | 14.32 | 6.66 | 10.51 | 14.14 | 0.76 | 0.73 |
|  | LMP | 0.08 | 0.11 | 19 | 0.12 | 262.21 | 0.13 | 0.94 | 1.02 | 0.78 | 0.66 | 1.23 | 0.68 | 6.45 | 10.54 | 14.28 | 6.71 | 10.62 | 14.22 | 0.81 | 0.75 |
|  | TTN | 0.08 | 0.11 | 19.35 | 0.11 | 244.12 | 0.15 | 0.94 | 1.02 | 0.78 | 0.63 | 1.39 | 0.67 | 6.45 | 10.39 | 13.71 | 6.69 | 10.51 | 13.99 | 0.73 | 0.7 |
| Chicken | FEW | 0.05 | 0.11 | 0.11 | 0.23 | 484.45 | 0.16 | 0.63 | 0.68 | 11.06 | 0.37 | 3.4 | 0.37 | 0.35 | 0.99 | 0.97 | 0.34 | 0.98 | 0.97 | 0.07 | 0.07 |
|  | EW36 | 0.05 | 0.11 | 0.1 | 0.22 | 509.34 | 0.16 | 0.62 | 0.67 | 10.56 | 0.37 | 3.34 | 0.37 | 0.35 | 0.93 | 1 | 0.34 | 1 | 1 | 0.07 | 0.07 |
|  | EW56 | 0.05 | 0.1 | 0.1 | 0.22 | 473.94 | 0.16 | 0.62 | 0.67 | 10.9 | 0.35 | 3.41 | 0.35 | 0.3 | 0.91 | 0.93 | 0.31 | 0.96 | 0.96 | 0.06 | 0.06 |
|  | EW80 | 0.04 | 0.09 | 0.08 | 0.21 | 438.16 | 0.15 | 0.62 | 0.66 | 10.78 | 0.28 | 3.33 | 0.29 | 0.25 | 0.75 | 0.79 | 0.26 | 0.75 | 0.75 | 0.04 | 0.05 |
| Sheep | AFL | 0.01 | 0.02 | 0.02 | 0.06 | 46.78 | 0.02 | 0.39 | 0.4 | 0.64 | 0.04 | 7.76 | 0.04 | 0.03 | 0.08 | 0.08 | 0.02 | 0.07 | 0.08 | 0.02 | 0.02 |
|  | TP | 0.01 | 0.02 | 0.02 | 0.07 | 46.95 | 0.02 | 0.37 | 0.38 | 0.64 | 0.04 | 8.26 | 0.04 | 0.03 | 0.1 | 0.1 | 0.03 | 0.09 | 0.09 | 0.02 | 0.02 |
|  | MLS | 0.01 | 0.02 | 0.02 | 0.08 | 47.81 | 0.02 | 0.4 | 0.41 | 0.66 | 0.04 | 8.61 | 0.04 | 0.04 | 0.1 | 0.1 | 0.03 | 0.1 | 0.09 | 0.02 | 0.02 |

Table S2. Runtime of different models based on SNP array data across four species. (minutes
